# Nutritional Metabolomics in Diet–Breast Cancer Relations: Current Research, Challenges, and Future Directions—A Review

**DOI:** 10.3390/biomedicines11071845

**Published:** 2023-06-27

**Authors:** Farhad Vahid, Kimia Hajizadeghan, Adeleh Khodabakhshi

**Affiliations:** 1 Nutrition and Health Research Group, Precision Health Department, Luxembourg Institute of Health, 1445 Strassen, Luxembourg; farhadvahid@outlook.com; 2 Department of Nutrition, Faculty of Public Health, Kerman University of Medical Sciences, Kerman 7616913555, Iran

**Keywords:** gut microbiota, multiple-omics approaches, genomics, transcriptomics, proteomics, personalized dietary recommendations

## Abstract

Breast cancer is one of the most common types of cancer in women worldwide, and its incidence is increasing. Diet has been identified as a modifiable risk factor for breast cancer, but the complex interplay between diet, metabolism, and cancer development is not fully understood. Nutritional metabolomics is a rapidly evolving field that can provide insights into the metabolic changes associated with dietary factors and their impact on breast cancer risk. The review’s objective is to provide a comprehensive overview of the current research on the application of nutritional metabolomics in understanding the relationship between diet and breast cancer. The search strategy involved querying several electronic databases, including PubMed, Scopus, Web of Science, and Google Scholar. The search terms included combinations of relevant keywords such as “nutritional metabolomics”, “diet”, “breast cancer”, “metabolites”, and “biomarkers”. In this review, both in vivo and in vitro studies were included, and we summarize the current state of knowledge on the role of nutritional metabolomics in understanding the diet–breast cancer relationship, including identifying specific metabolites and metabolic pathways associated with breast cancer risk. We also discuss the challenges associated with nutritional metabolomics research, including standardization of analytical methods, interpretation of complex data, and integration of multiple-omics approaches. Finally, we highlight future directions for nutritional metabolomics research in studying diet–breast cancer relations, including investigating the role of gut microbiota and integrating multiple-omics approaches. The application of nutritional metabolomics in the study of diet–breast cancer relations, including 2-amino-4-cyano butanoic acid, piperine, caprate, rosten-3β,17β-diol-monosulfate, and γ-carboxyethyl hydrochroman, among others, holds great promise for advancing our understanding of the role of diet in breast cancer development and identifying personalized dietary recommendations for breast cancer prevention, control, and treatment.

## 1. Introduction

Breast cancer stands for a substantial global health concern [1] and is the most commonly diagnosed cancer worldwide, with an estimated 2.26 million cases documented in 2020; it is one of the main justifications for cancer mortality among women, with more than 680 thousand deaths in 2020 (6.9% of global cancer-associated deaths) [1]. Despite advancements in investigations, diagnostics, and treatment, such shocking numbers persist. To reduce morbidity and/or mortality rates, understanding the exact etiology of breast cancer and designing/developing novel treatment/management approaches are necessary [2].

The role of diet and dietary patterns/habits in the etiology of breast cancer is undeniable [3]. For example, some diets/dietary patterns, such as a high intake of fructose and low intake of seafood and vegetables, and an inflammatory diet containing saturated fatty acids (SFA) and red and processed meat, are associated with increased risk of triple-negative breast cancer in in vivo and in vitro models [4,5]. On the other hand, the effect of some diets and food compounds such as polyphenols, antioxidant diets, and vitamins involved in one-carbon metabolism has been shown to reduce the risk of triple-positive breast cancer [3,6,7]. However, epidemiological evidence and randomized clinical trials for the relationship between dietary-related factors and breast cancer are robust; etiologic mechanisms are primarily uncertain and inconsistent [8]. A new approach to elucidating the relationship between nutrition and breast cancer is through novel omics science, especially metabolomics [9]. In brief, nutritional metabolomics is the study of diet-related metabolites to highlight diet and health outcome associations and may provide a method to seize exogenous dietary exposures more precisely and appraise endogenous markers mediating diet–cancer associations, or both [10,11,12]. Nutritional metabolomics allows us to consider multiple exposures related to diet and the metabolic procedures that they affect. Several studies used the nutritional metabolomics procedures in the lung, prostate, ovarian, and colorectal cancers [13] and colon adenoma [14] to determine serum metabolites that are associated with particular dietary exposures, such as intake of vegetables, fruit, nuts, meat, fish, fat, coffee, alcohol, and multivitamins. Findings from those studies regarding the role of diet-related biomarkers linked to metabolomics are able to potentially assist objective dietary explanation, abate errors/biases related to self-reported dietary patterns, agnostically assess metabolic pathways, and discern mechanistic mediators [15].

Some recent studies and meta-analyses comprehensively reviewed the association between diet–cancer relations [16,17,18,19,20,21], cancer metabolomics [22,23,24,25,26], nutritional metabolomics [27,28,29,30,31], or metabolomics methods and applications to epidemiology [32,33,34]. However, very limited (if any) studies to determine the function of nutritional metabolomics in diet–cancer research, to the best of our knowledge, have been conducted. Therefore, this review’s objective is to provide a comprehensive overview of the current research on the application of nutritional metabolomics in understanding the relationship between diet and breast cancer. This review aims to summarize current research, challenges, and future guidance for epidemiologic studies to clarify the role of nutritional metabolomics in breast cancer, identify the challenges associated with this field of study and propose potential future directions for research.

## 2. Methodology

### 2.1. Literature Search Strategy

To conduct a comprehensive review of the current research on nutritional metabolomics in diet–breast cancer relations, a systematic search of the literature was performed. The search strategy involved querying several electronic databases, including PubMed, Scopus, Web of Science, and Google Scholar. The search terms included combinations of relevant keywords, such as “nutritional metabolomics”, “diet”, “breast cancer”, “metabolites”, and “biomarkers”. The search was limited to articles published in English, and the date range was set from the databases’ inception up to the present. Additionally, the reference lists of the identified articles were manually scanned to identify additional relevant studies.

### 2.2. Inclusion and Exclusion Criteria

The articles retrieved from the literature search were screened based on predefined inclusion and exclusion criteria. Inclusion criteria consisted of studies focusing on nutritional metabolomics in the context of diet–breast cancer relations. Both in vivo and in vitro studies were included. Only original research articles, reviews, and meta-analyses were considered. Studies on identifying and quantifying metabolites related to dietary factors and breast cancer were included. Exclusion criteria comprised studies not directly related to the topic, non-English articles, conference abstracts, letters to the editor, and case reports.

### 2.3. Data Extraction

Data extraction was conducted independently by two reviewers. Relevant information was extracted from each included study, including study characteristics (e.g., study design, sample size), participant characteristics (e.g., age, sex), dietary factors assessed, metabolomics platforms used, metabolites identified, statistical methods employed, and key findings. Any discrepancies between the reviewers were resolved through discussion and consensus.

### 2.4. Data Synthesis and Analysis

The data extracted from the included studies were synthesized to provide an overview of the current research landscape on nutritional metabolomics in diet–breast cancer relations. A narrative synthesis approach was employed, highlighting each study’s key findings and methodological considerations. The studies were categorized based on the type of dietary factors investigated, such as macronutrients, micronutrients, dietary patterns, or specific food groups. The metabolomics platforms used were also described, including mass spectrometry and nuclear magnetic resonance spectroscopy. The main findings related to the identified metabolites and their associations with breast cancer risk or prognosis were summarized.

### 2.5. Ethical Considerations

As this review was based on the analysis of previously published studies, no ethical approval was required. All included studies had obtained appropriate ethical clearance and informed consent from their participants, as reported in their respective publications.

### 2.6. Limitations

The design of this review has some limitations. Firstly, the search strategy might not have captured all relevant articles, although efforts were made to minimize this possibility. Secondly, the inclusion of only English articles might have introduced language bias. Finally, the review focused on the existing literature up to the present date and, therefore, may not include the most recent studies.

## 3. Metabolomics and Nutritional Metabolomics: How Will They Be Helpful?

Supported by solid evidence, as there is a relationship between the human genome and nutrient intake, today, nutrition science concentrates more on personalized health-improving diets [35]. In the last decade, the technological progress in omics science has led to an efficient public health strategy and personalized nutrition [36]. Modern nutrition aims to use omics science, such as genomics, proteomics, and metabolomics to estimate a person’s response to particular food items or dietary patterns. By evaluating these reactions at the molecular level, it is possible to determine the most suitable diet or lifestyle for a special individual to improve people’s health or prevent and even treat diseases [37]. While genomics and transcriptomics have been widely used in nutrition studies, metabolomics is involved much less in nutritional research [38]. As mentioned, metabolomics is a field of omics science in which small molecules named metabolomes are measured [39,40] and are signs of gene and protein function. Therefore, disorders affecting gene and protein function can change metabolite abundance [41].

Metabolomics approaches include targeted and untargeted: Targeted metabolomics is defined as a quantitative analysis when absolute concentrations of selected metabolites are needed to be characterized to help extend biomarkers or test hypotheses. With untargeted metabolomics, unlike targeted metabolomics, there is less focus on specific metabolite identification and quantification, and it is often used for biomarker discovery studies [40,42]. Both of these approaches have their strengths and weaknesses, but as for quantifying bioactive compounds, there is increasing attention to quantitative metabolomics and targeted metabolomics in nutrition studies. A targeted approach is now broadly used in food composition analysis [43,44,45], the identification of biomarkers of food intake [28,29,40], and finding and monitoring metabolic/nutrient disorders and/or nutritional deficiencies [40]. In metabolomics, a wide range of tools is required. Three leading technologies are used primarily in metabolomics: gas chromatography–mass spectrometry (GC–MS) [46], nuclear magnetic resonance (NMR) spectroscopy [47], and liquid chromatography–MS (LC–MS) [48,49]. Each method broadly covers metabolites such as amino acids, lipids, sugars, organic acids, and biogenic amines. NMR is best at distinguishing metabolites with high quantities, while LC–MS and GC–MS are foremost at indicating lower amounts of metabolites [48,49,50]. Altogether, using several technologies expands the amount of metabolite coverage and types of samples [50].

In addition, nutritional metabolomics studies the metabolites indicating dietary consumption, gut absorption, digestion, biotransformation by tissues or host microbiota, and endogenous metabolites influenced by dietary intake [12,51]. In this study, we comprehensively reviewed nutritional metabolomics studies of breast cancer as epidemiologic studies to determine diet–cancer associations by measuring nutritional metabolites. In nutritional metabolomics, high-resolution chemical analysis together with statistical tools [52] has been used to determine chemicals in foods [43,53], recognize food byproducts in tissues and/or biofluids [12], determine macro–micronutrient imbalances [54,55], track biochemical reactions to dietary interventions [27], trace dietary patterns/habits [29], and instruct the advancement of diet therapies [56].

## 4. Critical Role of the Gut Microbiome in the Network

The gut microbiome refers to microorganisms existing in the gut and includes over a thousand bacterial species and, partly, fungi, viruses, archaea, and protists [57]. The microbiome describes a set of genomes that the microbiota (the bacterial population) own [58]. The microbiota contains up to 90% of human cells [59] and has been related to health and chronic disease [60,61]. The microbiota also has a significant role in the immune system, so improper proportions of healthy and harmful bacteria can lead to chronic conditions such as cancer [58,62,63]. When the ratios of microbiota species are suitable, they prepare energy by fermentation, synthesize vitamins, produce amino acids, and prevent chronic conditions and diseases [64]. Lifestyle factors such as diet and physical activity influence the complex relationship between gut microbiota and estrogen metabolism and affect both breast cancer recurrence and metastasis possibility [65]. Interventions that increase microbial diversity through dietary recommendations can affect health, particularly in patients with breast cancer [65]. The consumption of a poor diet containing a high level of processed meat, simple sugars, and salt and a sedentary lifestyle have a significant negative impact on the gut microbiota [65].

The estrobolome—the bacteria that are the subgroup of the microbiota responsible for estrogen metabolism and degradation—plays a significant role in breast cancer’s development and/or progression [66]. The primary source of energy for the estrobolome is fiber, and breast cancer risk is often associated with elevated levels of estrogen, which may be mitigated by a high-fiber diet that prevents breast cancer cells from accessing the fiber. However, recommending a diet high in fiber and polyphenols for individuals with breast cancer can reduce inflammation and improve breast cancer survival rates [67,68,69,70,71,72].

In addition, a study investigated the gut microbiome profile concerning tumor grade and stage, HER2 and ER/PR status, and selected risk factors in women diagnosed with breast cancer [73]. Women with early menarche and HER2+ compared with later menarche and HER2− breast cancers showed a significantly less diverse microbiome and a distinguished bacterial composition profile, including an abundance of Firmicutes. Women with breast cancer with an earlier menarche age (≤11) and with high total body fat (≥46%) had a lower diversity of gut microbiome [73].

In another study, recently diagnosed breast cancer postmenopausal women had a lower gut microbiota diversity and a divergent composition than those without breast cancer. Additionally, the breast-cancer-suffering women had a high level of urinary estrogens; however, it was independent of microbiota dissimilarities. The results highlight the fact that gut microbiota may have an effect on the risk of breast cancer, and perhaps it is through pathways independent of estrogen [74].

Several studies have shown that certain types of gut bacteria can affect the metabolism of dietary components, such as phytoestrogens, which are plant-derived compounds that can have either protective or harmful effects on breast cancer, depending on their concentration and form [75,76]. For instance, some gut bacteria can convert phytoestrogens to more active forms, stimulating breast cancer cell growth [77]. Conversely, other gut bacteria can metabolize phytoestrogens to less active forms, which can reduce breast cancer risk [77]. Furthermore, dysbiosis, a state of imbalance in the gut microbiome, has been associated with chronic inflammation, which can promote the growth and spread of cancer cells [78]. In addition, the gut microbiome can affect the absorption and bioavailability of nutrients from the diet, such as vitamins and minerals, which are important for maintaining healthy tissues and preventing cancer [79].

On the other hand, a special association between some bacteria and the decrease or increase in breast cancer has been shown. For instance, certain strains of Lactobacillus spp. bacteria in the gut have been associated with beneficial effects, including immune modulation and anti-inflammatory properties. Some studies suggest that specific Lactobacillus species may have a protective role against breast cancer development [80]. On the contrary, some studies have found an increased abundance of Bacteroides fragilis, a common gut bacterium associated with inflammation and immune dysregulation, in breast cancer patients, particularly in hormone receptor-negative breast cancer cases [81]. Overall, the gut microbiome is an important player in the diet and breast cancer network, and understanding its complex interactions with dietary components and immune function may provide novel avenues for the prevention and treatment of breast cancer.

## 5. Current Research on Food-Related Metabolites and Their Role in Breast Cancer

### 5.1. Piperine

Studies suggest that piperine, an exogenous active alkaloid found in black and long pepper, has an inverse association with breast cancer risk [82]. Several studies offered beneficial properties for piperine, including antioxidant [83,84], anti-inflammatory [82,83,84], anticonvulsive, antimutagenic, antimycobacterial, and anti-cancer activities, including the inhibition of angiogenesis and increased cell apoptosis [82], especially in breast cancer models [85,86,87]. Some studies suggest that piperine may have anti-cancer attributes, such as the capacity to hinder the growth and spread of cancer cells. For example, a study found that piperine inhibits breast cancer cell growth in a laboratory setting [88]. Another study suggested that piperine may effectively suppress the growth of breast cancer stem cells, which are reported to play a role in the development and recurrence of breast cancer [85]. However, it is important to note that these studies were conducted in vitro (in a laboratory setting), and further research is needed to determine if piperine could be an effective treatment for breast cancer in humans. Overall, while the preliminary results of studies are promising, more studies are needed to fully understand the potential benefits and risks of using piperine as a natural therapy for breast cancer.

### 5.2. Acetyl Tributyl Citrate (ATBC)

Acetyl tributyl citrate (ATBC) is a plasticizer to phthalates [89], usually functioning as a food additive and contact material [89,90]. The relocation of ATBC from food packaging into food has been observed in wrapped cake, cheese, peanut-comprising cookies, and microwaved soup [90]. Recent studies showed the potential biological activity of ATBC on tissue growth [91] and a potential interruption of ovarian function in female mice due to exposure to ATBC [92]. However, the available evidence of the relationship between ATBC exposure and breast cancer risk is limited and conflicting. Some animal studies have suggested that ATBC may act as an endocrine disruptor and promote the growth of breast cancer cells, while other studies have found no significant effects [93,94]. There is currently no conclusive evidence linking ATBC exposure to an increased risk of breast cancer in humans. However, given the potential health risks associated with exposure to endocrine-disrupting chemicals like ATBC, it is important to minimize exposure whenever possible. Overall, more studies are needed to better understand the potential link between ATBC and other plasticizers with breast cancer risk.

### 5.3. Metabolite of Alcohol

Alcohol consumption is among the confirmed modifiable dietary risk factor for increased risk of breast cancer with solid documentation, which has been classified by the International Agency for Research on Cancer (IARC) as a Group 1 carcinogen [95,96]. Acetaldehyde (a metabolite of alcohol, a toxic and carcinogenic substance that can cause DNA damage and impair cellular function) is rapidly converted to acetate, a less toxic substance, by the enzyme aldehyde dehydrogenase (ALDH) in the liver. However, some individuals have a genetic variation that results in lower levels of ALDH activity, resulting in an accumulation of acetaldehyde in the body when alcohol is consumed. This buildup of acetaldehyde has been associated with an increased risk of breast cancer [97]. Furthermore, alcohol consumption can also affect the levels of other hormones in the body, including estrogen, which can stimulate the growth of breast cancer cells [98]. Alcohol has been shown to increase circulating estrogen levels in both premenopausal and postmenopausal women, which may contribute to the increased risk of breast cancer associated with alcohol consumption. In addition, alcohol consumption can also impair the function of the immune system, which plays a crucial role in breast cancer development and progression [99]. Chronic alcohol consumption has been associated with increased levels of inflammation and oxidative stress, promoting the growth of cancer cells and spreading [6]. Alcohol intake may increase circulating levels of steroid hormones, which could affect susceptibility to transform or promote cancer growth [98]. Several mechanisms have been suggested for considering alcohol as a serious risk factor for breast cancer: a randomized alcohol-feeding study in postmenopausal women reported that alcohol increased serum dehydroepiandrosterone sulfate [100], resulting in circulating androgens and estrogens [101,102].

In addition, prospective epidemiologic studies reported that other androgens, dehydroepiandrosterone sulfate, and estrogens are significantly related to postmenopausal breast cancer [103,104] and alcohol [105]. Hence, alcohol may increase the risk of postmenopausal breast cancer via the enhanced production of estrogens, androgens, and their metabolites. Metabolites related to alcohol, such as a-hydroxyisovalerate (a hydroxy-fatty acid derivative) and ethyl-glucuronide (a metabolite of ethanol breakdown), irrelevant to androgen metabolism, are also linked to ER+ breast cancer. Their associations remained unaffected regarding controlling androgen-pathway metabolites, representing that alcohol may act by androgen-independent mechanisms to raise breast cancer risk [106]. In addition, acetaldehyde, as a metabolite of alcohol, can bind to DNA and cause genetic mutations, which may also contribute to the development of breast cancer. Acetaldehyde may also impair DNA repair mechanisms, increasing the risk of breast cancer [96]. Overall, the metabolites of alcohol, particularly acetaldehyde, can contribute to the increased risk of breast cancer associated with alcohol consumption.

### 5.4. 2-Amino-4-cyano Butanoic

2-amino-4-cyano butanoic acid is a non-proteinogenic alpha-amino acid (2-aminobutanoic acid), so-called alpha-amino-gamma-cyano butanoic acid, substituted at position four by a cyano group and an aliphatic nitrile [107]. It may be made from butyrate, a short-chain fatty acid mainly produced from fibers’ fermentation via colon bacteria [107]. Butyrate might be a crucial nutrient for colonocyte function and also a primary protective factor against breast cancer [108]. It has beneficial effects on intestinal homeostasis and energy metabolism. Butyrate improves intestinal barrier function, mucosal immunity, and anti-inflammatory properties [109]. Results show that butyrate may be considered an interesting inhibitor for breast cancer progression by inhibiting MCF-7 cell proliferation and promoting apoptosis in breast cancer [110]. The production and effects of butyrate are affected by diet, especially dietary fibers (legumes, beans, peas, soybeans, fruits, nuts, cereals, and whole grains) and fat consumption [111]. A few studies have investigated the effects of 2-amino-4-cyano butanoic on breast cancer cells, but the results have been mixed. Some studies have suggested that 2-amino-4-cyano butanoic may be able to stimulate apoptosis in breast cancer cells, while others have found no significant effects [112,113]. To date, no large-scale studies have examined the relationship between 2-amino-4-cyano butanoic and breast cancer in humans. Therefore, it is challenging to draw any conclusions about the potential role of 2-amino-4-cyano butanoic in breast cancer prevention or treatment. It is worth noting that while 2-amino-4-cyano butanoic may have some potential as an anti-cancer agent, more research is needed to fully understand its mechanisms of action and potential side effects.

### 5.5. Alpha-Tocopherol

Alpha-Tocopherol is the main circulating tocopherol isoform that prevents the oxidation of polyunsaturated fatty acid (PUFA). The main dietary sources are nuts and seeds, vegetable oils (wheat germ oil, safflower oil, and sunflower oil), and green leafy vegetables (spinach, kale, and Swiss char). In addition, the main dietary sources for γ-tocopherol are margarine and vegetable oil [114], and, for δ-tocopherol, castor oils, soy, and margarine [115]. Experimental studies demonstrate that α- and γ-tocopherols may increase apoptosis and reduce cell proliferation, two highlighted cancer markers [116,117,118]. However, some prospective studies indicate conflicting results, highlighting the (non)insignificant association between breast cancer and circulating α- or γ-tocopherol [119,120,121,122,123,124,125], a significant positive association with γ-tocopherol [126], and a significant inverse association with α-tocopherol [127]. While it has been suggested that alpha-tocopherol may have potential anti-cancer effects, research on its relationship with breast cancer has yielded mixed results. Some studies have suggested that alpha-tocopherol intake may decrease the risk of developing breast cancer [128]. For example, a study found that a higher intake of alpha-tocopherol is associated with a reduced risk of breast cancer among postmenopausal women [129]. However, other studies have found no significant association between alpha-tocopherol intake and breast cancer risk [130,131]. For example, a study found no evidence that alpha-tocopherol supplementation reduced breast cancer risk among women with a high risk of developing the disease [132]. Furthermore, some studies have suggested that high doses of alpha-tocopherol supplements may actually increase the risk of developing breast cancer [129,133]. For example, a study found that high-dose vitamin E supplementation was associated with an increased risk of developing breast cancer among postmenopausal women [134]. As some studies suggest that higher intake may be protective, others suggest that high doses of supplementation may increase the risk of developing the disease; more research is needed to fully understand the relationship between alpha-tocopherol and breast cancer.

### 5.6. Metabolites of Fat

In breast cancer investigations, metabolites of fat such as butter-related caprate (10:0), a medium-chain SFA (saturated fatty acids); dairy fat-related 10-undecenoate (11:1n–1), an odd-carbon MUFA (monounsaturated fatty acids); fried food-related 2-hydroxyoctanoate, a hydroxy fatty acid; and dessert-related g-CEHC are associated with a risk of ER+ breast cancer [135].

The association between dietary fat and postmenopausal breast cancer has been discussed due to contradictory findings in the literature. Such conflicts can show etiologic relations differ in the type of dietary fat, various degrees of measurement error in estimating fat intake [136,137,138,139], or both, indicating strong positive links with SFAs than other fats [140]. Nutritional metabolomics will somewhat solve these concerns by providing measurable biomarkers that contain a full range of dietary fat. Inflammation, increased estrogen synthesis in adipose tissue, and modifications in various physiologic processes (e.g., immune function, breast cell response to growth factors, and tumor suppression) are proposed mechanisms for the effect of dietary fat on breast carcinogenesis [140]. In addition, an analysis of urine reported that levels of caprate (10:0) were higher in mice with breast tumors compared with control groups [141].

In addition, phospholipid lysophosphatidylcholine (LPC) correlates with a lower risk of breast cancer, while a higher level of phosphatidylcholine (PC) correlates with an increased risk of breast cancer [142]. Lysophosphatidylcholine acyltransferase 1 (LPCAT1) converts LPC to PC, and it is overexpressed in breast cancer with poor prognosis [143], which demonstrates that a higher level of PC is associated with a high risk of breast cancer. In addition, a study conducted a metabolomics analysis in a large cohort of women and identified several metabolites that were positively associated with breast cancer risk, including sphingomyelin (d18:1/18:0), phosphatidylcholine (16:0/18:1), and lysophosphatidylcholine (C17:0) [13,144]. Another study reported that high levels of circulating SFA, particularly myristic acid (14:0), are correlated with an increased risk of breast cancer [135]. Moreover, a study examined the association between diet-dependent acid load and breast cancer risk. It concluded that a higher dietary acid load is associated with an increased risk of breast cancer. The study also found that specific metabolites, including certain fatty acids and amino acids, were positively associated with breast cancer risk [145].

On the other hand, one type of fat metabolite that has been studied extensively in relation to breast cancer is the omega-3 fatty acid, docosahexaenoic acid (DHA). Studies have shown that DHA can suppress breast cancer cell growth and migration and may also enhance the effectiveness of certain chemotherapeutic drugs used to treat breast cancer [146,147]. On the other hand, high levels of omega-6 fatty acids, such as arachidonic acid, have been associated with increased breast cancer risk and poor prognosis [148,149]. Another important class of fat metabolites that has been linked to breast cancer is adipokines. Adipokines are hormones secreted by adipose tissue and play a role in regulating metabolism and inflammation [150]. It has been shown that leptin, an adipokine, promotes breast cancer cell growth and survival, while adiponectin, another adipokine, has been found to have antitumor effects [151,152]. In addition, the gut microbiome also plays a role in regulating fat metabolism and can influence breast cancer risk. Certain gut bacteria produce short-chain fatty acids (SCFAs) as a byproduct of dietary fiber fermentation. For example, it has been shown that SCFAs have anti-inflammatory effects and may help protect against breast cancer [153]. The association between fat metabolites and breast cancer is complicated, and future research is needed to fully understand the involved mechanisms. However, evidence suggests that maintaining a healthy balance of different types of fats in the diet and promoting a healthy gut microbiome may help reduce breast cancer risk and improve outcomes for those diagnosed.

### 5.7. Metabolites of Protein

Combining metabolomics and proteomics analysis can give us a more comprehensive understanding of breast cancer. The proteomics data particularly distinguished 29 upregulated proteins, such as GSS, GOT1, GPX3, and LDHB, and two downregulated proteins, including GC and dipeptidyl peptidase 4 (DPP4), in breast cancer [154]. Among the 31 specific proteins, LDHB, GSS, GOT1, and GPX3 were scrupulously related to metabolites and collectively contributed to various major metabolic pathways, such as the arginine biosynthesis pathway; arginine and proline metabolism; glycolysis or gluconeogenesis; alanine, aspartate, and glutamate pathway; glutathione metabolism; and cysteine and methionine metabolism [155]. Altered arginine and asparagine levels correlate with breast cancer [156,157]. Reducing dietary asparagine, or the blocking of asparagine synthetase, reduces breast cancer metastasis [156].

Additionally, it has been suggested that breast cancer cells, in order to maintain the life activities of tumor cells, absorb a lot of glutamate from blood circulation [158]. Studies have shown that glutamate, via activating the alpha-amino-3-hydroxy-5-methyl-4-isoxazolepropionic acid receptors (AMPAR), as well as promoting breast cancer cell invasion and migration through the activation of the MAPK pathway, could accelerate tumorigenesis [159,160]. In breast cancer, under normoxic conditions, glutamate was also found to enhance the activity of HIF1α [161]. In addition to these findings, amino acid metabolism could also play a significant role in breast cancer. Some studies have identified alterations in amino acid metabolism in breast cancer cells, including changes in the levels of certain amino acids and their metabolites [162]. For example, one study found that breast cancer cells have increased uptake and utilization of certain amino acids, such as glutamine and serine, to support their growth and survival [163]. Other studies have identified changes in the expression levels of enzymes involved in amino acid metabolism, such as argininosuccinate synthase, which is involved in arginine metabolism and has been found to be upregulated in breast cancer cells [163]. The findings suggest that targeting amino acid metabolism is probably a potential strategy for treating breast cancer. Further understanding of how this metabolism influences breast cancer is important.

### 5.8. Metabolites of Carbohydrate

Cancer cells are highly dependent on glycolysis [164]. Moreover, the metabolites involved in energy metabolism pathways, such as glycolysis, are altered in breast cancer [165]. These metabolites are ATP, acetyl-CoA, NADPH, and lactate [166]. Moreover, cancer cells can adapt well to increased lactate production and survive in acidic microenvironments [167]. This leads to an accumulation of lactate in the tumor microenvironment, which can result in acidification of the surrounding tissue [167]. Despite this acidic environment, cancer cells can adapt and survive by upregulating certain mechanisms, such as proton transporters and ion channels, to help maintain intracellular pH and avoid cell death [168].

Additionally, some studies have suggested that lactate itself may have signaling functions that contribute to tumor growth and progression [169]. In addition, higher lactate levels are associated with lower survival rates [170]. In addition, high levels of blood glucose, insulin, and insulin-like growth factor 1 (IGF-1) have been associated with an increased risk of breast cancer [171]. These findings have shown that carbohydrate metabolism’s metabolites play a vital role in breast cancer growth and progression. Understanding the roles and mechanisms by which these metabolites can affect breast cancer will be significant.

## 6. Summary of Current Research Linking Diet-Related Metabolites and Breast Cancer

In a study of 113 metabolites related to diet, three were linked with altogether breast cancer risk: γ-carboxyethyl hydrochroman (γ-CEHC, a γ-tocopherol derivative), 4-and rosten-3β,17β-diol-monosulfate (an androgen), and caprate (10:0), a saturated fatty acid [135]. Nineteen metabolites were significantly associated with ER+ breast cancer: twelve alcohol-associated metabolites, including seven α-hydroxyisovalerate and androgens; three vitamin E (tocopherol) derivatives (e.g., γ-CEHC); caprate (10:0); and fried food-associated 2-hydroxyoctanoate [135]. Moreover, a significant positive association was reported between the intake of liquor and piperine and a decreased risk of breast cancer [135]. According to the study [135], prediagnostic serum levels of metabolites related to animal fats, alcohol, and vitamin E were strongly and moderately related to the risk of ER+ breast cancer.

In another study, ten metabolites were positively associated with the risk of breast cancer in premenopausal women, especially glycerol, NAC, ethanol, and histidine [172]. This study shows various metabolite associations with breast cancer by menopausal status [172]. Additionally, in another study, a lower level of piperine and higher levels of 2-amino-4-cyano butanoic acid (a metabolite linked to microbiota metabolism), acetyltributylcitrate ATBS, pregnenetriol sulfate (a steroid sulfate) were seen in plasma from women who subsequently developed breast cancer [107]. In this study, ATBC was positively associated with coffee intake; pregnenetriol sulfate was positively associated with alcoholic beverage consumption; piperine was positively associated with alcohol consumption and with a Westernized dietary pattern; and 2-amino-cyano butanoic acid was negatively associated with an intake of biscuits and cake [107]. The undisclosed compositions were related to several dietary exposures, such as the intake of salty products, pasta and cereals, processed meat, citrus fruit, tomatoes, and press-cooked cheese [107]. Of the 14 diet-related compounds, piperine and six unknown compounds were inversely associated with breast cancer [107]. 2-amino-4-cyano butanoic acid, ATBC, and pregnenetriol sulfate were positively related to breast cancer [107].

Moreover, a positive association between coffee intake and plasma ATBC could show contaminant movement from plastic cups into the coffee. Milk added in coffee may assist this relocation as ATBC is disposed to migrate into protein liquids, such as skim milk solution [173]. Increased plasma levels of ATBC in women with breast cancer could also have other sources that this study has not detected. In addition, the positive association between piperine and the “Western” dietary pattern is because of the consumption of several foods such as sauces, industrial cheese, processed meat, and poultry-containing piperine as a food additive or via pepper as part of a Westernized dietary pattern or intake with alcoholic beverages [173].

Another study found a direct association between wine consumption and piperine in the serum of female twins [174]. However, the origin of circulating piperine is not limited to Western dietary patterns and can also result from adding black pepper to food. A steroid sulfate hormone from the progestin family, so-called pregnenetriol sulfate, is positively associated with both breast cancer risk and alcohol intake [174]. In another study, positive associations between alcohol consumption and various serum steroids were highlighted, especially pregnenediol sulfate, which is associated with an increased risk of breast cancer [100,175]. Moreover, there is a strong association between incremented levels of sex steroids and postmenopausal breast cancer risk [176].

An interventional study investigating postmenopausal women revealed that the serum level of dehydroepiandrosterone (DHEA) sulfate, a precursor to androstenedione, testosterone, and, eventually, estradiol and estrone, is increased after alcohol intake [177]. Furthermore, the pregnenetriol sulfate driven by 17-hydroxy-pregnenolone, which loses its side chain, can produce DHEA [177]. Other factors, including body mass index (BMI) and lactation, could influence the level of sex steroids [178]. Furthermore, it has been shown that ATBC may alter endogenous steroid hormone metabolism by activating human and rat steroids and xenobiotic receptors (SXRs) [179]. The study concluded an inverse association between concentrations of arginine, asparagine, and phosphatidylcholine (PC) ae C36:3 with breast cancer risk, while acylcarnitine C2 was positively associated with disease risk [179].

Additionally, a negative association between PC ae C34: 2, C36:2, and C36:3 concentrations and C38:2 with adiposity were found, and a positive association with total and saturated fat intake was observed [180]. PC ae C36:2 was also negatively associated with alcohol intake and positively associated with two scores reflecting adherence to a healthy lifestyle. The concentration of asparagine was negatively associated with adiposity. Further, there was a positive association between acetylcarnitine and age [180].

The transport of fatty acids from the cytosol into the mitochondria can be facilitated by acylcarnitine C2. An increased concentration is a marker for lipid oversupply and upregulated fatty acid oxidation [181]. In cancer cell biology, lipid oversupply by providing essential raw materials needed to produce new cells enhances cancer cell proliferation [182]. It is suggested that perhaps chronic lipid oversupply supplies energy and nutrients to growing tumors.

In some studies [154,183], the glutamine and glutamate metabolic pathways; the alanine, aspartate, and glutamate pathway; and the arginine biosynthesis pathway were the important metabolic pathways in breast cancer pathogenesis. Additionally identified was a panel of 47 metabolites, such as glutamate, sphingomyelins, and cysteine, which could be effective diagnostic methods for diagnosing breast cancer [154,183].

The study [154,183] highlighted that intervention studies would provide a better understanding of the origin of its variations and relation to food and their association with the risk of breast cancer and that the finding should be repeated in other independent observational investigations, and a potential causal relationship could be investigated through cellular mechanistic studies.

In addition, the food metabolites, breast cancer type, and main involved cellular protein/receptor are summarized in Table 1.

## 7. Challenges

Nutritional metabolomics has the potential to provide insights into the mechanisms underlying the link between diet and breast cancer, as well as identify potential biomarkers for diagnosis and treatment. However, several challenges need to be addressed in order to fully realize the potential of nutritional metabolomics in diet–breast cancer relations:

1. Complexity of diet: One of the biggest challenges in nutritional metabolomics is the complexity of the human diet. The human diet contains thousands of different compounds, and the interactions between these compounds are not well understood. This makes it challenging to identify specific metabolites that are associated with breast cancer risk;
2. Inter-individual variability: There is a high degree of inter-individual variability in how people metabolize dietary compounds. This can make it difficult to identify consistent associations between dietary metabolites and breast cancer risk across different populations;
3. Timing of exposure: The timing of dietary exposure may be critical in determining its effect on breast cancer risk. However, most studies rely on self-reported dietary intake, which may not accurately reflect the timing and duration of exposure;
4. Small sample sizes: nutritional metabolomics studies often have small sample sizes, which can limit statistical power and increase the risk of false positive or false negative results;
5. Validation and replication: The validation and replication of findings in independent populations is critical in nutritional metabolomics research. However, the lack of standardized metabolite identification and quantification methods can make it challenging to replicate findings across different studies;
6. Data integration: Integrating data from different omics platforms (e.g., genomics, transcriptomics, and proteomics) can provide a more comprehensive understanding of the mechanisms underlying the link between diet and breast cancer. However, integrating data from different platforms can be challenging due to differences in data quality, processing, and analysis methods. Overall, these challenges highlight the need for rigorous study design, standardized methods, and collaboration across disciplines in order to advance our comprehension of the role of dietary metabolites in breast cancer risk.

## 8. Future Directions

The use of nutritional metabolomics in understanding the relationship between diet and breast cancer is a relatively new field. One future direction for nutritional metabolomics in the study of diet–breast cancer relations is the identification of specific metabolites or metabolic pathways associated with increased or decreased risk of breast cancer. This can help in the development of personalized dietary recommendations for individuals at high risk of breast cancer. Another direction is investigating the role of gut microbiota in the metabolism of dietary components and their impact on breast cancer risk. The gut microbiota also play a crucial role in the metabolism of nutrients and bioactive compounds, and recent research has suggested that alterations in the gut microbiota may contribute to breast cancer development. Additionally, integrating multiple-omics approaches, such as genomics, proteomics, and transcriptomics, with nutritional metabolomics can provide a more comprehensive understanding of the complex interactions between dietary factors and breast cancer risk. Overall, the application of nutritional metabolomics in the study of diet–breast cancer relations holds great promise for advancing our comprehension of the role of diet in breast cancer development and identifying personalized dietary recommendations for breast cancer prevention and treatment.

## Figures and Tables

**Table 1:** Food metabolite/compound, breast cancer type, and main involved cellular protein/receptor.

Breast Cancer Type	Metabolite/Compound	Main Protein/Receptor	Ref.
Ductal carcinoma in situ (DCIS)	Piperine	HER2	[13,135,184]
	Alpha-Tocopherol	HER2	[185]
	Metabolites of fat (Palmitic acid)	CD36	[186]
	Metabolites of protein (e.g., Insulin-like growth factor 1)	IGF-1 Receptor	[187]
Invasive ductal carcinoma (IDC)	Acetyl tributyl citrate (ATBC)	Estrogen Receptor (ER)	[188]
	Metabolite of alcohol (e.g., Acetaldehyde)	Estrogen Receptor (ER)	[189]
	2-amino-4-cyano butanoic	Aromatase	[188]
	Alpha-Tocopherol	HER2	[190]
Invasive lobular carcinoma (ILC)	Metabolite of alcohol (Acetaldehyde)	Estrogen Receptor (ER)	[191]
	Alpha-Tocopherol	HER2	[190]
	Metabolites of fat (Palmitic acid)	CD36	[192,193]
	Metabolites of protein (e.g., Insulin-like growth factor 1)	IGF-1 Receptor	[194]
	Metabolites of carbohydrate (e.g., Glucose)	Hexokinase 2 (HK2)	[195]
Triple-negative breast cancer (TNBC)	Piperine	HER2	[85]
	Metabolite of alcohol (e.g., Acetaldehyde)	Estrogen Receptor (ER)	[196]
	Alpha-Tocopherol	HER2	[197]
	Metabolites of fat	CD36	[198,199]
	Metabolites of protein (e.g., Insulin-like growth factor 1)	IGF-1 Receptor	[200,201]
	Metabolites of carbohydrates (e.g., Glucose)	Hexokinase 2 (HK2)	[202]
Hormone receptor-positive (HR+)	Metabolites of fat (e.g., Palmitic acid)	CD36	[199,203]
	Metabolites of protein (e.g., Insulin-like growth factor 1)	IGF-1 Receptor	[204]
	Metabolites of carbohydrate (e.g., Glucose)	Hexokinase 2 (HK2)	[205]
Hormone receptor-negative (HR-)	Piperine	HER2	[13,135,184]
	Metabolite of alcohol (e.g., Acetaldehyde)	Estrogen Receptor (ER)	[206]
	2-amino-4-cyano butanoic	Aromatase	[107]
	Alpha-Tocopherol	HER2	[190]
	Metabolites of fat (e.g., Palmitic acid)	CD36	[207]
	Metabolites of protein (e.g., Insulin-like growth factor 1)	IGF-1 Receptor	[201]
	Metabolites of carbohydrate (e.g., Glucose)	Hexokinase 2 (HK2)	[208]

## Data Availability

Not applicable.
